# Integrative genomic analysis of salivary duct carcinoma

**DOI:** 10.1038/s41598-020-72096-2

**Published:** 2020-09-14

**Authors:** Youngwook Kim, Sanghoon Song, Miran Lee, Teresa Swatloski, Joon Ho Kang, Young-Hyeh Ko, Woong-Yang Park, Han-Sin Jeong, Keunchil Park

**Affiliations:** 1 Department of Health Science and Technology, Samsung Advanced Institute for Health Science and Technology, Sungkyunkwan University School of Medicine, Seoul, 06351 Republic of Korea; 2 Samsung Biomedical Research Institute, Samsung Medical Center, Seoul, 06351 Republic of Korea; 3 Theragen Bio Insitute, Suwon, 16229 Republic of Korea; 4 Biomolecular Engineering Department, University of California, Santa Cruz, CA 95066 USA; 5 Department of Pathology, Samsung Medical Center, Sungkuyunkwan University School of Medicine, Seoul, 06351 Republic of Korea; 6 Samsung Genome Institute, Samsung Medical Center, Seoul, 06351 Republic of Korea; 7 Graduate School of Cancer Sciecne and Policy, National Cancer Center, Goyang, 10408 Republic of Korea; 8 Department of Otorhinolaryngology-Head and Neck Surgery, Samsung Medical Center, Sungkuyunkwan University School of Medicine, Seoul, 06351 Republic of Korea; 9 Division of Hematology/Oncology, Department of Internal Medicine, Innovative Cancer Medicine Institute, Samsung Medical Center, Sungkyunkwan University School of Medicine, Seoul, 06351 Republic of Korea

**Keywords:** Cancer, Computational biology and bioinformatics, Oncology, Cancer genomics

## Abstract

Salivary duct carcinoma (SDC) is one of the most aggressive subtypes of salivary gland cancers. Conventional chemotherapy and/or radiation have shown only limited clinical efficacy in the treatment of recurrent or metastatic SDC. Currently, clinically approved targeted-therapeutics are not generally applicable except in very limited cases, and there exists a strong need for the development of treatment against this unique tumor type. To further interrogate genomic features of SDC, we have conducted multi-omic profiling of the SDC to describe the genomic alterations prevalent in this disease. Whole-genome sequencing, whole exome-sequencing and transcriptome sequencing were performed on a discovery cohort of 10 SDC samples. Targeted genomic profiling was performed in additional 32 SDC samples to support the findings obtained from the original discovery cohort. The cancer cohort was characterized by an average mutation burden of 85 somatic exonic mutations per tumor sample. The cohort harbored a mutational signature of BRCA and APOBEC/AID. Several genes, including *TP53*, *RB1*, *SMAD4*, *HRAS*, *APC*, *PIK3CA* and *GNAQ* were recurrently somatically altered in SDC. A novel fusion gene, generated by genomic rearrangement, *MYB-NHSL1*, was also noted. Our findings represent a significant layer in the systematic understanding of potentially clinically useful genomic and molecular targets for a subset of recurrent/metastatic SDC.

## Introduction

Salivary duct carcinoma (SDC) is a rare and aggressive histological subtype of salivary gland cancers. The recurrence rate of SDC is high and median overall survival is about 3 years^1^. In general, conventional chemotherapy and radiation have only limited efficacy for the metastatic SDC^2^.

SDC shares morphological and histological similarities to invasive ductal carcinoma of the breast^3^. This resemblance of SDC to breast ductal carcinoma led to an investigation of the hormonal receptor and human epidermal growth factor receptor 2 (HER2)/neu expression status in SDC. Although estrogen and progesterone receptor are rarely over-expressed in SDC, androgen receptor expression is commonly observed^4,5^. Androgen deprivation therapy in combination with radiotherapy demonstrated clinical benefits in some preliminary cases^6,7^. Among salivary gland cancers, overexpression and gene amplification of *HER2* were specifically associated with SDC^8,9^. Based on the expression pattern of these potentially actionable targets, some studies have reported a positive treatment outcome with anti-HER2 trastuzumab in combination with radio-chemotherapy^10–12^.

Previous genetic analyses focused on specific cancer genes and little data exists on the genomic landscape of SDC^13,14^. More recently, high-throughput molecular characterization of SDC has been reported^15,16^, which provides information on the disease entity with an unbiased genomic scale. However, multi-dimensional genomic characterization of SDC, including whole-genome sequencing data, remains to be completed.

Building on the previous genomic works in SDC, we report the results of whole-genome sequencing, whole exome-sequencing and RNA sequencing analysis of 10 high-grade SDC cases. Targeted sequencing and copy number analysis were also conducted in 32 additional comparable samples. Present findings in conjunction with other genomic analysis of the disease can help in gaining understanding of molecular mechanism underlying SDC.

## Materials and methods

### Clinical samples

Written informed consent forms were acquired from patients who agreed to donate their tissue samples. Following Institutional Review Board (IRB) approval, tumor and matched normal specimens were collected from patients with SDC at Samsung Medical Center (Seoul, Korea) during the years 2015 to 2018. All subjects submitted the written informed consent for the use of their clinical information and tissue samples.

For discovery phase SDC samples (designated sd01–sd10), surgical or biopsy tissue samples were snap frozen at the time of acquisition and were stored in liquid nitrogen until they were further processed for genomic analysis. Hematoxylin and eosin-stained and immune-stained tumor slides were reevaluated to confirm the original diagnosis of SDC by a pathologist (YHK), who has comprehensive experience (over 10 years) of salivary gland pathology. For validation phase SDC samples (designated 01–32), tissue samples were obtained from the same center and subjected to targeted sequencing implemented in the sequencing facility at Samsung Medical Center (Seoul, Korea) for Ion-Torrent Ampliseq panel sequencing. Clinical information was collected and immuno-staining of marker proteins, including androgen receptor, was performed to confirm the pathological diagnosis (Supplementary Methods S1). The diagnosis of SDC was based on the criteria described on WHO classification of head and neck tumors^17^. The most typical histologic changes are high grade carcinoma resembling that of breast, showing large ducts with comedonecrosis, cribriform or Roman bridge-like feature. Histologic variants such as micropapillary, sarcomatoid and mucin-rich variants were identified. Some cases showed focal preexisting pleomorphic adenoma.

### Preparation of DNA and RNA samples

DNA was extracted from snap-frozen tissue and/or whole blood using the DNeasy Blood and Tissue Kit (Qiagen, Hilden, Germany). The concentration of DNA samples was quantified by nanodrop and PicoGreen assay (Thermo Fisher Scientific, Waltham, MA, USA). RNA was obtained using the RNeasy Mini or Micro kit (Qiagen, Germany) and quantified with nanodrop or RiboGreen assay (Thermo Fisher Scientific). The integrity and quality of nucleic acid samples were analyzed by BioAnalyzer (Agilent Technologies, Santa Clara, CA, USA) (Supplementary Methods S2).

### Targeted sequencing of validation set

Ion Torrent AmpliSeq Cancer Hotspot Panel v2 (Life Technologies, Carlsbad, CA, USA) was used to sequence cancer mutation hotspot sites in more than 50 oncogenes and tumor suppressor genes. For multiplex PCR amplification, 10 ng of DNA, quantified by Qubit Fluorometer (Thermo Fisher Scientific), was used and the custom Ion AmpliSeq panel was applied with the Ion AmpliSeq Library kit 2.0 according to the manufacturere’s instructions. Resulting amplicons were treated with FuPa Reagent to partially digest the primers and phosphorylate amplicons. The amplicons were then ligated to the Ion XpressTM Barcode Adapters (1–96 Kit) and template preparation was performed with the Ion One-TouchTM System using an Ion OneTouchTM 200 Template Kit v2 DL. Sequencing was performed on Ion 316 chips using the Ion PGMTM 200 Sequencing Kit according to the manufacturere’s instructions. Raw signal data were analyzed using Torrent Suite v.4.0.2 (Life Technologies) with the Torrent Mapping Alignment Program. Variant calling was performed using the Torrent variant Caller 4.0 software and was annotated with Oncotator and SnpEff^18^.

### Nanostring analysis of copy number alteration in validation set

To detect somatic copy number alterations (SCNAs) in SDC, a panel of customized gene probes was designed and subsequently analyzed on the NanoString nCounter platform. Custom NanoString probes adopted in the current study consist of the following 21 genes: *AURKA*, *CCND1*, *CCNE1*, *CDK4*, *CDK6*, *CDKN1A*, *CDKN2A*, *EGFR*, *ERBB2*, *ERBB3*, *FGFR1*, *FGFR2*, *IGF1R*, *KLF5*, *KRAS*, *MDM2*, *MET*, *MITF*, *MYC*, *PIK3CA*, *TNIK*. For the NanoString nCounter assay, 600 ng of genomic DNA was hybridized with the custom designed codes for 18 h at 65 °C and processed according to the manufacturer’s instruction^18^. Data were normalized to the invariant control probes and to positive and negative controls in each hybridization reaction.

### Massively parallel sequencing and data availability

Whole-genome and whole-exome sequencing data were generated at Macrogen, Inc. (Seoul, Korea), DNA Link, Inc. (Seoul, Korea) and Theragen Etex Co. Ltd (Suwon, Korea). RNA sequencing data was generated at ChunLab, Inc (Seoul, Korea) (Supplementary Table S1).

### Mutation analysis

The matching IDs of tumor and normal samples of each patient were genomically confirmed with VerifyBamID. Raw sequencing data were aligned to the hs37d5 genome build with Burrows–Wheeler Aligner. Indel realignment, base quality score recalibration and removal of duplicated reads were performed with the Genome Analysis Toolkit version 4.

Single nucleotide variants (SNVs) were detected by MuTect2. SNVs were further filtered using Korean SNP database to ensure removal of erroneous false-positive calls. Mutations were independently scrutinized by manual inspection using Integrative Genomics Viewer (IGV) v2.3 (broadinsistute.org/igv). Insertions and deletions were identified by Indelocator.

### Network analysis

Ordered list of somatic mutations observed from SDC samples (10 in discovery cohort + 32 in validation set) was used as input in the enrichment analysis of g:Profiler toolkit (https://biit.cs.ut.ee/gprofiler) to identify statistically enriched molecular processes implicated in the pathogenesis of SDC.

### Tumor purity and ploidy estimation

To determine the tumor purity of each sample, several algorithms, including ABSOLUTE (broadinstittue.org/cga/absolute), FACETS^19^ and PyLOH^20^ was used to estimate purity and ploidy values from each sample.

### Somatic copy number alteration analysis

SCNA data were generated and analyzed by FACETS^19^ and BIC-seq2 (https://compbio.med.harvard.edu/BIC-seq/). The segmentation data obtained from FACETS and BIC-seq2 was used as input data for GISTIC 2.0^21^ to determine the statistical significance of the SCNAs in the SDC samples and to generate curated amplification/deletion profile of the corresponding SCNA events.

### Structural rearrangement analysis

Large-scale DNA structural change was analyzed by employing the DELLY algorithm^22^. Fusion gene analysis using RNA sequencing data was done in parallel to comparatively analyze translocation events in the tumor samples^23–25^. Structural rearrangements were depicted with RCircos package.

### Gene expression analysis

RNA sequencing data was aligned and processed by in-house custom pipelines. Differential gene expression analysis was performed with data partly processed by TopHat and Cufflinks^26^. Tumor map analysis was performed following reprocessing of raw RNA sequencing data using the same pipeline that produced the tumor map profile (https://tumormap.ucsc.edu). Immune signature analysis was conducted with CIBERSORT^27^.

### Conference presentation

This was presented in 2018 ASCO meeting 10.1200/JCO.2018.36.15_suppl.6083.

### Ethics approval and consent to participate

Written informed consent forms were acquired from patients who agreed to donate their tissue samples. Following Institutional Review Board (IRB) approval (IRB no. 2015-06-132, Samsung Medical Center), tumor and matched normal specimens were collected. All methods were carried out in accordance with relevant guidelines and regulations.

### Consent for publication

All subjects submitted the written informed consent for the use of their clinical information and tissue samples.

## Results and discussion

### Genomic portrait of SDC

To determine the genomic landscape of somatic alterations in SDC, massively parallel paired-end sequencing was conducted on 10 discovery cohort samples. These samples were scrutinized with independent round of pathology review to ensure their correct classification as SDC, a classic example of which is illustrated in the figure (Supplementary Fig. S1). We additionally performed next-generation targeted sequencing on 32 SDC samples to corroborate with data obtained from the discovery cohort. The median age of patients with SDC in the present study was 64 years (range 39–85). The median overall survival and median recurrence-free survival were 40 and 25 months, respectively (Table 1 and Supplementary Table S2).

**Table 1.** Clinical information of subjects (N = 42).

	No	Percentage (%)
Age at diagnosis (years, median, [range])	64 [39–85]	
**Gender**		
Male	35	83.3
Female	7	16.7
**Primary tumor site**		
Parotid gland	36	85.7
Submandibular gland	4	9.5
Sublingual or minor gland	2	4.8
**Smoking status**		
Never smoker	22	52.4
Former smoker	5	11.9
Current smoker	12	28.6
NA	3	7.1
**T classification**		
T1	3	7.1
T2	20	47.6
T3	9	21.4
T4	10	23.8
**N classification**		
N0	22	52.4
N1	2	4.8
N2	15	35.7
N3	1	2.4
NA	2	4.8
**AR staining**		
Positive	25	59.5
Negative	14	33.3
NA	3	7.1
**Recurrences**		
No + NA	24	57.1
Local/regional recur	4	9.5
Distant metastasis	14	33.3

*NA* no available information, *AR* androgen receptor.

The discovery cohort was analyzed with multiple-level genomic profiling, including whole-genome, whole-exome and transcriptome sequencing from fresh-frozen tumor and matched normal samples. For whole-exome sequencing, the mean sequencing depth was 129.6X for tumor DNA and 129.3X for normal DNA. More than 98.6% of the target region of both tumor and normal DNA was covered at least 20 times. Inter-sample contamination of all the samples was minimal (less than 0.2%), as determined by Contest^28^. We detected a median of 85 nonsynomymous somatic mutations in and around the coding region of the genome per tumor, which corresponds to 1.7 somatic nonsynonmous mutations per megabase. This frequency of non-synonymous mutations was comparable to that of breast and kidney cancers. Overall, the mutational burden of the SDC cohort is placed in the lower third of all solid tumors^29^.

We further conducted massively parallel-targeted sequencing of additional 32 archived SDC samples to probe the mutational status of a selected gene set. We applied a recurrence-based selection method with several covariates included in the statistical consideration to select a subset of genes that are implicated in the pathogenesis of SDC. The composite mutational profile of SDC samples demonstrates that *TP53*, *RB1*, *SMAD4*, *APC*, *PIK3CA*, *HRAS* and *GNAQ* genes have demonstrably recurrent somatic mutations in SDC. Additional cancer genes that are annotated in the COSMIC database include mutations in *ERBB2*, *FLT3*, *ERBB3*, *ERBB4* and *MET* genes.

**Figure 1:**
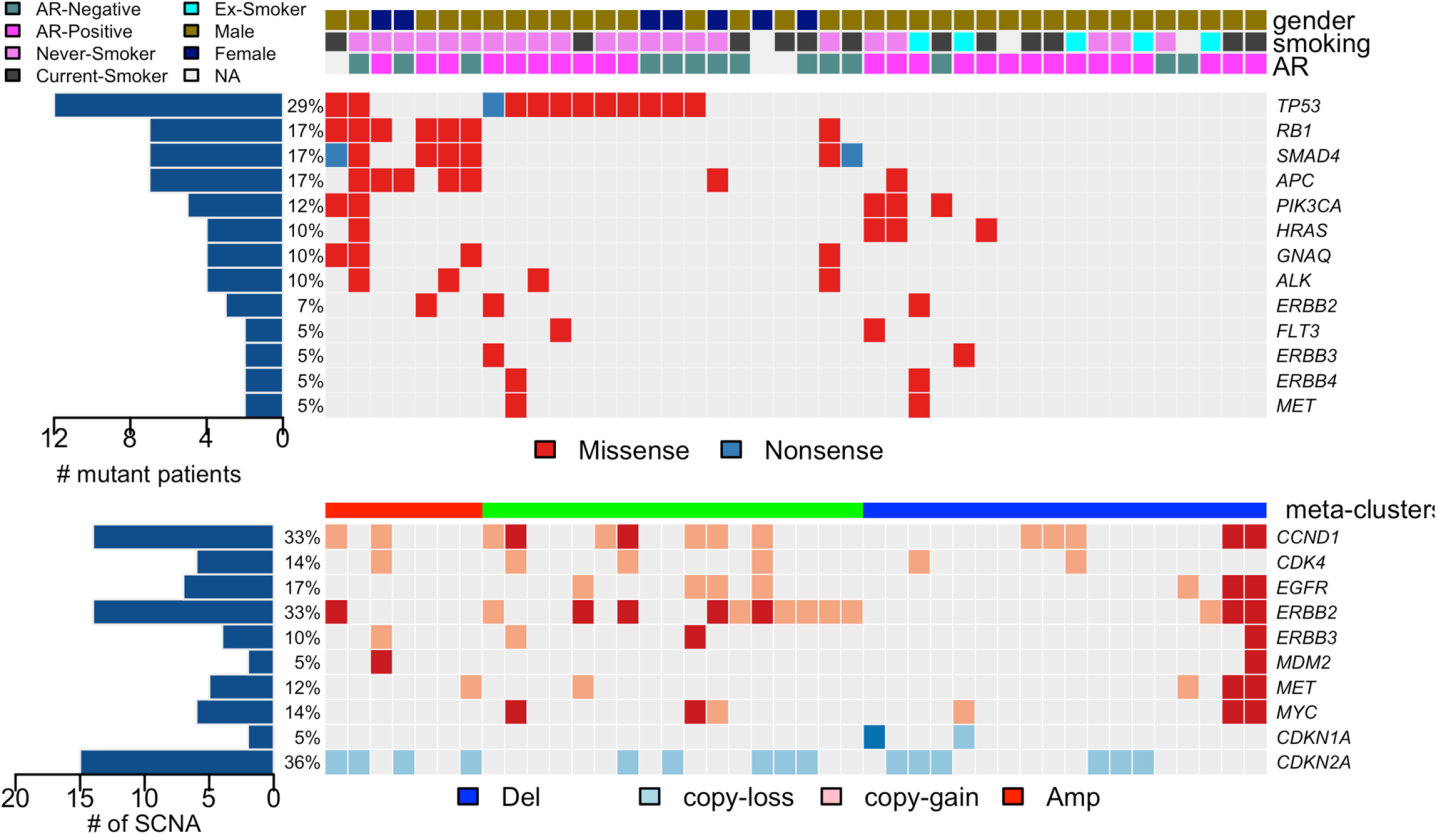
Clinico-pathological characterization and genetic aberrations across 42 salivary duct carcinomas. The clinic-pathological features were depicted in the top panel. The first row indicates gender, the second row smoking-status and the third row Androgen Receptor staining. The panel in the middle is the heatmap representation of individual mutations present in 42 salivary duct cancer samples in association with information from the top panel. It shows the mutational types in a given sample and in a given gene in 2-dimensional matrix format. (Left) Percentage of mutations in each gene in the cohort. (Right) List of recurrently mutated genes. The panel in the bottom is the heatmap of somatic copy number alterations (SCNAs) of SDC samples in association with panels in the top and in the middle. Significant SCNAs are shown. SCNAs were categorized into 4 different classes, depending on the degree of SCNAs; deletion, copy-loss, copy-gain and amplification.

Hierarchical clustering of the cohort based on the combination of clinical information, mutational pattern and somatic copy number variations resulted in three clinico-mutational clusters (Fig. 1). Cluster 1 was predominantly represented by co-occurring somatic mutations in *RB1*, *SMAD4*, *GNAQ1* and *APC* (Fisher’s exact test, *p* = 0.004). Cluster 2 exhibited predominant *TP53* mutations with an exclusivity relationship with the top 4 other most frequently recurrent mutations in SDC (Fisher’s exact test, *p* = 0.002). Cluster 3 was mostly mutation-silent with several visible somatic copy number alterations. It is noteworthy that smoking status does not reveal any particular association with specific molecular subtypes and suggests that smoking does not have direct influence on the mutagenesis of SDC, in stark contrast with the more direct impact smoking imposes on upper aero-digestive tract cancer types^29^.

**Figure 2:**
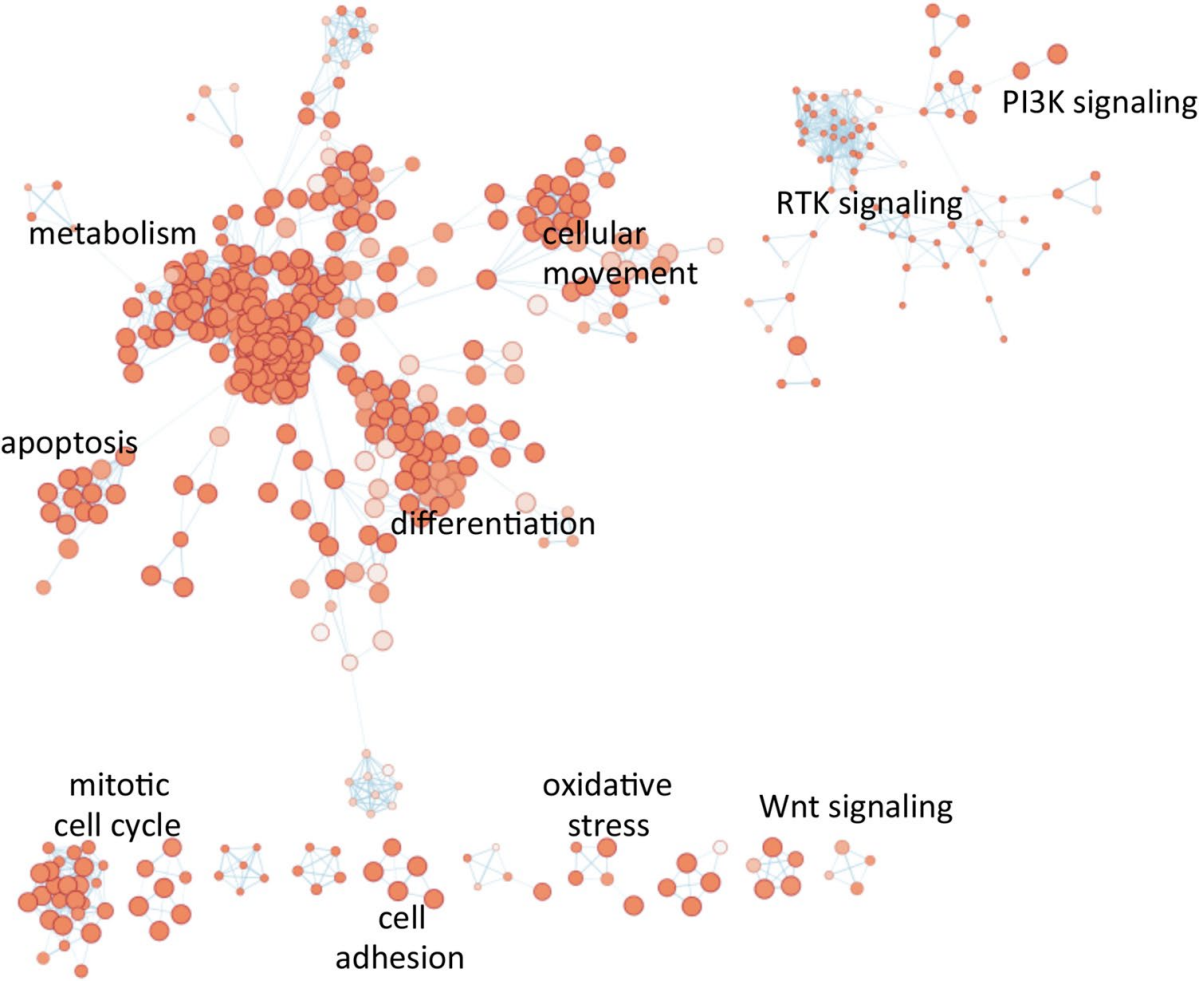
Functional profiling and network analysis of SDC somatic mutations. Ordered list of somatic mutations frequently observed in SDC were used as input for characterizing the gene list. Molecular processes functionally enriched in SDC were annotated in the clustered nodes of the network, the analyses of which is described in the method section.

Ordered list of cancer genes observed in SDC revealed core processes implicated in discrete functional categories. The molecular processes and pathways significantly enriched in SDC include receptor tyrosine kinase (RTK) signaling, PI3K signaling and Wnt signaling (Fig. 2). Other processes were mitotic cell cycle, apoptosis, and oxidative stress responses. Metabolic processes and cellular movement processes were also included in significantly enriched processes, implicating their association with pathogenesis of high-grade SDC.

### Mutational signature analysis of SDC

**Figure 3:**
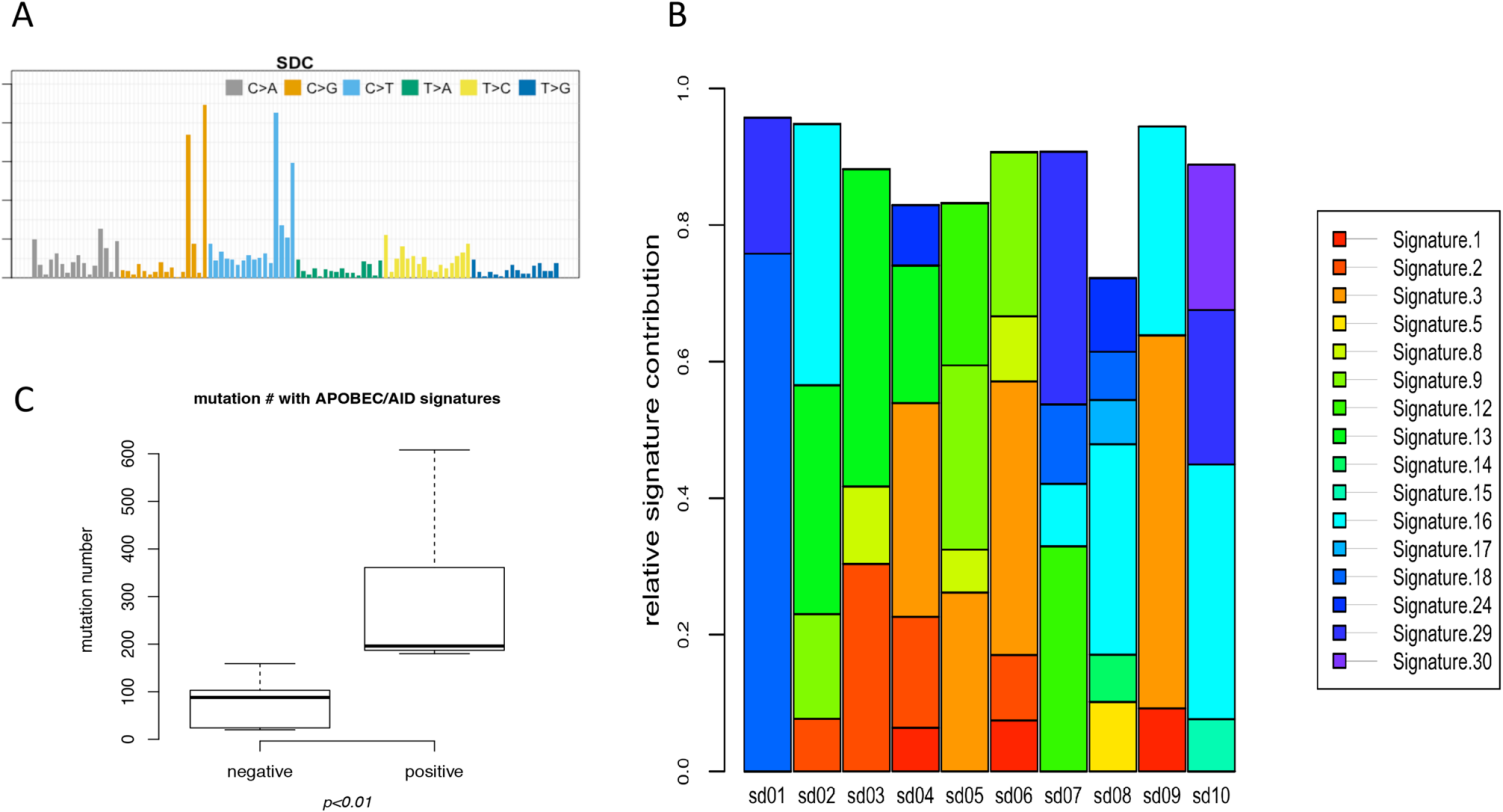
Mutational signature analysis of SDC. (**A**) Mutational signature analysis of SDC cohort. Point mutations of SDC samples were aggregated to form a set of ‘ensemble SDC mutations’. This ensemble mutation set was used in mutation signature analysis to decipher representative signatures in SDC cohort. (**B**) Contribution of mutation signatures to each SDC sample. Somatic mutations identified from genomic sequencing of SDC samples were subjected to mutation signature analysis per sample. Patterns of 1937 single-nucleotide mutations in 10 SDC samples were analyzed and the contribution of each signature to the mutagenesis of SDC samples are shown. X-axis is the name of sample and y-axis is the relative contribution of mutation signature normalized per sample. (**C**) Comparison of mutation rate between samples with and without APOBEC/AID signatures. The number of somatic mutations in the exonic region of SDC between groups with and without APOBEC/AID signatures (signature 2,13/9) were compared and presented in the box-plot.

To decipher the underlying biological processes operative in generating the mutational profile of SDC, we performed mutation signature analyses^30^ on the SDC sample cohort (Fig. 3A). The somatic mutations in the SDC cohort were predominantly attributable to a few mutational signatures^31–33^ (Fig. 3B), including COSMIC signature 3, 16, 2 and 13. Signature 3 has been found in a subset of samples (sample IDs: sd04, sd05, sd06 and sd09) and is strongly associated with mutations in *BRCA* genes and their related components. In conjunction with recent in-depth genomic association study of signature 3 causality^34^, we detected somatic alterations in *BRCA2* (sd05) and in *PALB2* (sd04) in samples possessing strong mutation signature 3. Signature 16 was detected in multiple SDC samples (sample ID: sd02, sd07, sd08, sd09 and sd10). This signature has been shown to exhibit an extremely strong transcriptional strand bias for T > C mutations at ApTpN context, with T > C mutations occurring almost exclusively on the transcribed strand (https://cancer.sanger.ac.uk/cosmic/signatures). Signatures 2 and 13 strongly contributed to the mutational profile of samples 02, 03, 04 and less so to sample 06. Signatures 2 and 13 usually co-occur and are generated by actions of the AID/APOBEC family of cytidine deaminases. In line with this, the expression level of *APOBEC3A* was markedly upregulated in the sd04 sample. Signature 9, attributable to AID, also contributed to the mutational profile of multiple samples. Samples with more mutational burden were generally enriched with somatic mutations stemming from AID/APOBEC-related mutagenesis (Fig. 3C, Wilcoxon rank-sum test, *p* = 0.03). Analysis of germ-line DNA did not reveal any associable *BRCA* gene mutations in the cohort. Other signatures that contribute to multiple samples of the SDC cohort include signatures 18 and 29. The etiology of signature 18 is currently unknown, while signature 29 has been associated with tobacco chewing habits (https://cancer.sanger.ac.uk/cosmic/signatures). In conjunction with the previous observations that clinical smoking history was co-segregated with relatively silent somatic mutational pattern in SDC, the smoking signature was not detected in the current SDC cohort. Clustering of SDC samples based on mutational signatures produced 3 different clusters (Supplementary Fig. S2).

Consistent with the observation that sd04 sample overexpressed *APOBEC3A* with AID/APOBEC signature, this sample contains highly clustered somatic mutational pattern in two genomic loci (Supplementary Fig. S3).

### Copy number alterations

Somatic copy number alterations (SCNAs) of individual SDC samples were inferred from whole-genome sequencing data by incorporating allele-specific copy number estimation corrected for tumor purity, ploidy and heterogeneity^19,35^ (Supplementary Fig. S4). We subsequently applied GISTIC 2.0 analysis to the discovery cohort in order to identify measurably recurrent peaks of amplification and deletion^21^. Statistically significant amplification of chromosome 17q12, an amplicon containing *ERBB2*, was identified (Supplementary Fig. S5). We further extended SCNA analysis of SDC by incorporating targeted nanostring analysis of the validation set. The composite SCNA pattern demonstrated that a cluster containing co-amplifying genes, such as *MYC*, *CCND1* and *ERBB3* exist in virtual exclusivity with deleted *CDKN* genes. Analysis of DNA structural rearrangements showed the involvement of structural variation in many different ways in the pathogenesis of SDC. Inactivation of tumor-suppressor gene *CDKN2A* by highly clustered somatic DNA rearrangement was observed in one sample (SD05) (Supplementary Fig. S6), demonstrating multiple ways of activating and inactivating genes related to cell cycle in SDC.

### Fusion genes

**Figure 4:**
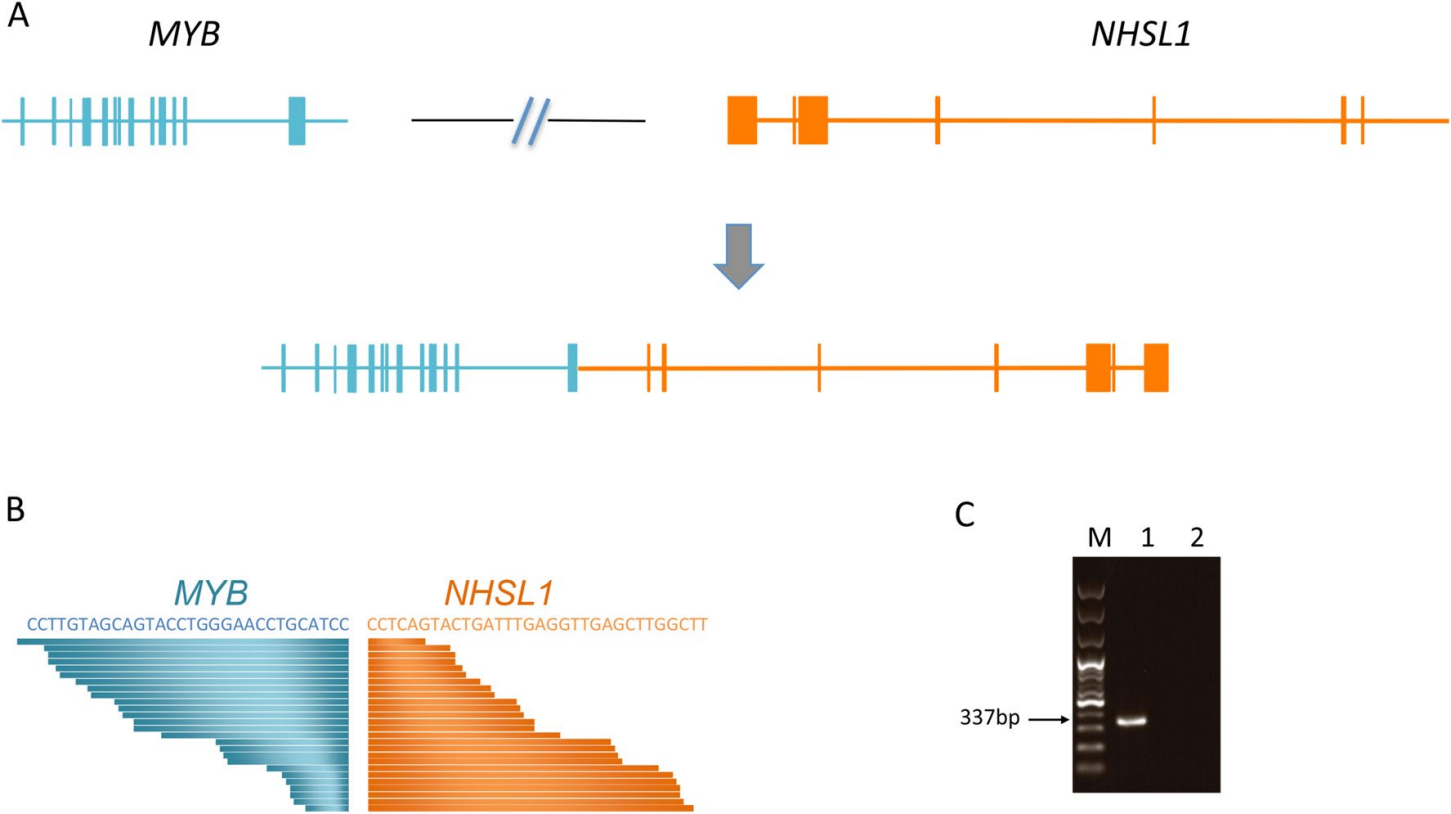
*MYB-NSHL1* fusion gene in SDC. (**A**) Representation of DNA rearrangements in *MYB-NHSL1* fusion SDC samples. *MYB* and *NHSL1* genes are located in the same chromosome 6, separated by around 3.2 megabase. The complex genomic DNA rearrangement event involving chromosome 6 relocates *MYB* and *MHSL1* gene in reverse orientation, producing the *MYB-NHSL1* fusion gene. (**B**) *MYB-NHSL1* fusion identified from RNA-sequencing. 60 split-reads that span the *MYB-NHSL1* junction are depicted. (**C**) *MYB-NHSL1* rearrangement-specific PCR reaction from genomic DNA derived from SDC patients. Sequencing chromatogram of a patient spanning the fusion junction. Sample 1 is the SDC sample harboring *MYB-NSHL1* and sample 2 is a control SDC sample.

Paired-end transcriptome sequencing data was generated for all the discovery SDC cohort samples. We initially processed these data to generate expression profiles at the transcript and gene levels. Further data analysis with a composite of independent pipelines^23–25^ collectively identified numerous fusion candidates at the RNA level. Fusion events frequently occurring in other subtypes of salivary gland cancers, including *MECT1-MAML2*^36^ in mucoepidermoid carcinoma, *MYB-NF1B*^37^ in adenoid cystic carcinoma, *ETV6-NTRK3*^38^ in mammary analogue secretory carcinoma of salivary glands and *EWSR1-ATF1*^39^ in hyalinizing clear-cell carcinoma of salivary glands, were not detected in the SDC cohort. From the SDC cohort, however, we identified a case involving the novel *MYB* and *NHSL1* fusion in sample sd03 (Fig. 4A,B). Detailed analysis of the fusion event demonstrated that the last exon at the 3′ end of *MYB* gene is fused with the 2nd exon of *NHSL1* gene. We conducted gDNA PCR analysis and confirmed the fusion point of the two genes by Sanger-sequencing (Fig. 4C). This fusion event generates an in-frame fusion between the two transcripts. The omission of the last exon of the *MYB* gene was reported to stabilize the *MYB* transcript by removing the 3′-UTR region, which contains several conserved target sites for miR-15a/16 and miR-150^40^. This fusion was also accompanied by concomitant marked overexpression of both the fused transcripts (Supplementary Fig. S7). The sample containing the *MYB-NSHL1* fusion gene was AR positive for immune-histochemical staining and pathological re-examination confirmed its original stratification as salivary duct carcinoma (Supplementary Fig. S8). In addition, cytokeratin 7 was expressed in the excretory duct component of SDC tumors. However, the expression of p63 (or p40) was not detected, suggesting that the basal or myoepithelial components were already replaced with extensive growth of carcinoma cells. Expression of Myb also confirmed our finding of MYB-NSHL1 gene fusion in this specimen.

Recent advances in immuno-therapy of cancer have brought keen interest in the composition of immune cells in tumor microenvironment. With the bulk RNA-sequencing data, components of immune cells in SDC cancerous tissue compartment were decomposed and assessed^27^ (Supplementary Fig. S9). In SDC, there usually exist high proportion of dendritic and macrophage cells and a few tumors exhibited a high proportion of CD8-positive T cells.

### SDC is mapped to breast cancer in RNA expression tumor map

Morphologically, SDC is similar to ductal carcinoma of the breast. The two disease entities share some molecular and clinical commonalities, such as *ERBB2* expression. Thus, it is hypothesized that breast cancer and SDC may be similar at the molecular level. To decipher possible molecular overlaps between these two anatomically separate tumor types, we processed transcriptome sequencing data obtained from SDC to identify cancer expression patterns that most closely resembles that of SDC^41^. Initial mapping efforts segregated the SDC discovery cohort samples mostly into breast cancer attribute. 8 out of 10 SDC samples were mapped to breast cancer cluster, supporting the morphological observation of similarities between the two disease entities (Supplementary Fig. S10). Closer examination of SDC tumor map data showed that these SDC samples were mapped to HER2 subtype of BRCA (breast carcinoma) (3 out of 8), LumA (luminalA) subtype (4 out of 8) and 1 basal subtype, suggesting certain level of heterogeneity and variability between SDC sample sets. 2 samples out of 3, which were mapped to HER2 subtype, harbored *Her2* gene focal amplification, consistent with their assignment to the specific BRCA subtype.

### Comparative mutational analyses of SDC

Using the annotated set of genomic alterations in this SDC cohort, we interrogated the association between somatic variations and clinical parameters. Most of the major mutated genes did not show statistically significant associations with overall survival (Supplementary Figs. S11–12). However, a few cases of somatic mutations and copy number alterations were statistically significantly associated with poor survival. The clinical and biological significance of these alterations in genes with apparent prognostic value in SDC needs further molecular analyses and independent validation in other SDC sets.

Given that discrepancies of genomic alteration between different ethnicities are present even in the same cancer type, the mutational alteration profiles of this cohort were compared with other target-sequencing based cohorts, consisting mostly of Caucasian patients^42,43^. The tumor mutational burden of other studies is more than twofold higher than in this study. This is most likely due to over-estimation of mutational burden in targeted sequencing approaches, as these studies employ a small amount of paraformaldehyde fixed samples, requiring more extensive amplification with a conspicuous lack of matched normal tissues. The mutational burden reported in this study is more in line with another unbiased genomic approach to this cancer type^16^, further confirming that the tumor mutational burden reported in the current study is a more accurate measure of a mutational profile in SDC. Tumor mutational burden has been shown to be an important biomarker to predict responses to immune checkpoint inhibitors^44–46^, and accurate measurement of tumor mutational burden is presumed to have significant impact in the selection of oncological precision medicine. Although SDC, in average, is characterized by relatively low non-synonymous mutation rate, some patients in the cohort are presented with exceedingly high tumor mutational burden and this subset of patients should be considered for cancer immunotherapy specifically tailored for them.

Consistent among all the studies conducted so far is a similarly high rate of mutations in *TP53*, mutations in *HRAS* and mutations in *PIK3CA*^44–46^. These studies also concordantly demonstrated high rate of *ERBB2* gene amplification. However, there are some notable differences between the datasets. The mutation of *RB1* was not placed in the top 10 mutated genes in other studies, whereas it was the second most frequently mutated gene in the current study (Fisher’s exact test, *p* = 0.02). *SMAD4* mutations were also frequent in the current study (17%), whereas the mutation rate in Caucasian samples was marginal (Fisher’s exact test, *p* = 0.02). The discrepantly higher *RB1* mutation rate in East-Asian cohort samples has been reported in other types of cancers^47,48^ and efforts to understand the ethnic differences may lead to better understanding of the tumor pathogenesis mechanisms uniquely prevalent in East-Asians.

## Conclusions

SDC is a rare and highly malignant salivary gland cancer with few currently proven target therapeutics, and it has almost no predictive molecular markers. Under current therapy settings, the majority of unresectable tumors become treatment-resistant within a short period of time.

Our data demonstrate that SDC is a heterogeneous disease at the genomic level. Although SDC samples share many histo-morphological features between themselves, they possess very diverse genomic alterations when assessed at the genome level, with the most frequent mutated genes harboring less than half of altered patterns. Closer examination of the genomic profile also suggests that many of SDC samples possess at least one target gene with direct or related targeted inhibitors currently available. Thus, it is important to translate knowledge obtained from genomic analysis of SDC samples into clinical cancer sequencing combined with precision oncology clinical trials.

Our accurate measure of tumor mutational burden and de-convolution of mutational signatures contributing to SDC mutational profile demonstrate a strong correlation between tumor mutational burden and operative mutational signatures. Given the importance of overall tumor mutational burden, it will be interesting to monitor the associable impact of SDC cancer signatures with clinical responses to immune-oncology therapeutics. Some SDC cancers exhibited marked overexpression of *ERBB2*, as previously reported. In clinical settings, there are patient candidates for employing targeted therapeutics against ERBB2^12^ and this could serve as one of clinical predictive biomarkers in stratifying SDC patients for their responsiveness to particular target therapeutics. There are also cases where SDC patients respond to androgen deprivation therapy^6,7,49^ and it will be critically important to monitor the responses to all of these potential target therapeutics in the context of tumor heterogeneity. Since SDC has a relatively low incidence rate, there exist only a handful of prospective clinical trials, most of which are single-arm clinical trial studies. However, these studies have already illuminated potential of such biomarker-driven target therapeutics: in one of phase II clinical trials, trastuzumab and docetaxel combination therapy in HER2-positive SDC patients showed clinical response rate of 70.2% with median progression-free survival and overall survival times 8.9 months and 39.7 months, respectively^12^. These examples demonstrate that targeted molecular screening approaches, including HER2 and AR, should be considered as a routine molecular testing in clinical practice settings.

Along with the ongoing efforts to accumulate our precise genomic understanding of the disease, a lack of in vitro and in vivo models for SDC tumor is another bottleneck for the functional study of SDC. Recent progress and developments in establishing patients-derived cancer cells will make functional screening more feasible and to critically test certain aspects of several findings obtained from the unbiased genomic analysis.

## Supplementary information

Supplementary Information.

## Data Availability

All the sequencing dataset described can be accessed through designated public database.

## Abbreviations

SDC: Salivary duct carcinoma
RTK: Receptor tyrosine kinase
HER2: Human epidermal growth factor receptor2
LumA: Luminal A
SCNA: Somatic copy number alterations
BRCA: Breast carcinoma
