# Supplementary material for: Integrative genomic analysis of salivary duct carcinoma
Source: Sci Rep. 2020 Sep 14;10:14995. doi: 10.1038/s41598-020-72096-2 (PMC7490354; doi:10.1038/s41598-020-72096-2)
Supplement: Supplementary file 1 — Supplementary Information. [file 41598_2020_72096_MOESM1_ESM.pdf]

Integrative Genomic Analysis of Salivary Duct Carcinoma

Youngwook Kim[1,6,7], Sanghoon Song[3], Miran Lee[2], Teresa Swatloski[4] , Joon Ho Kang[1,6], Young-Hyeh Ko[5], Woong-Yang Park[6], Han-Sin Jeong[8]*, Keunchil Park[2,9]*

[1] Department of Health Science and Technology, Samsung Advanced Institute for Health Science and Technology, Sungkyunkwan University School of Medicine, Seoul, 06351, Republic of Korea

[2] Samsung Biomedical Research Institute, Samsung Medical Center, Seoul, 06351, Republic of Korea

[3] Theragen Bio Insitute, Suwon, 16229, Republic of Korea

[4] Biomolecular Engineering Department, University of California, Santa Cruz, CA, USA, 95066

[5] Department of Pathology, Samsung Medical Center, Sungkuyunkwan University School of Medicine, Seoul, 06351, Republic of Korea

[6] Samsung Genome Institute, Samsung Medical Center, Seoul, 06351, Republic of Korea

[7] Graduate School of Cancer Sciecne and Policy, National Cancer Center, 10408, Republic of Korea

[8] Department of Otorhinolaryngology-Head and Neck Surgery, Samsung Medical Center, Sungkuyunkwan University School of Medicine, Seoul, 06351, Republic of Korea

[9] Division of Hematology/Oncology, Innovative Cancer Medicine Institute, Samsung Medical Center, Sungkyunkwan University School of Medicine, Seoul, 06351, Republic of Korea

* Corresponding author:

H.S.J. hansin.jeong@samsung.com; K.P. kpark@skku.edu

Supplementary Materials

Supplementary Methods S1: Biospecimen collection and clinical data:

Tumor samples were collected from SDC patients with surgical resection. Each fresh-frozen tumor sample was collected with corresponding adjacent normal tissue or matched blood samples. Each case was reviewed by two or more independent pathologists at Samsung Medical Center

For sequencing studies, we initially collected 30 SDC tissue samples with matched adjacent normal tissue or blood samples from the same individual. 14 samples passed initial quality control screening for DNA and RNA sequencing studies. Of these, 4 samples were further excluded on the basis of their low tumor cellularity. Final 10 samples were sequenced at multiple levels, including whole-genome, whole-exome and transcriptome sequencing.

Additional 37 samples were further screened from FFPE tissue archives to identify their somatic mutations with Ion-torrent Ampliseq assay. Copy number alterations were determined by nCounter Nanostring assay. Out of the 37 samples, 5 samples were removed from the final analysis for their quality control issues. Excluding these cases, the final set of composite SDC mutations consists of 10 samples in the discovery cohort analyzed with multi-dimensional sequencing and 32 additional validation samples.

Supplementary Methods S2: Transcriptome sequencing

Total RNA from each tumor sample was prepared using RNeasy mini kit (Qiagen, Germany). Transcriptome sequencing was performed on 10 SDC discovery cohort samples with adequate amount of RNA (1ug) and integrity (RIN> 7.0, rRNA ratio > 1.5). RNA library for sequencing on the illumine Hiseq 2000 was generated according to the protocol for the Illumina TruSeq sample preparation kit. Analysis of transcriptome-sequencing data was performed with

RNA-fusion was detected by running three different algorithms in parallel. These include CHIMERASCAN, DEFUSE and FUSIONMAP. Fusion transcripts supported by more than two pipelines with adequate reliance-score were selected as primary-fusion candidates.

Supplementary Table 1: Specimen and assay summary

Assay	Samples
Whole genome sequencing	10 pairs
Transcriptome sequencing	10 pairs
Whole exome sequencing	10 pairs
Ion-torrent ampliseq	32 pairs
Nanostring nCounter	32 pairs

Supplementary Table 2: Clinical and pathological data of the SDC cohort (N=10)

Discovery cohort

Number	Sex	Age	Smoking.status	Site	Pathology	Comments
sd01	M	43y9m	No	Parotid gland	SDC	Squamous differentiation
sd02	M	84y11m	No	Parotid gland	SDC	Poorly differentiated carcinoma
sd03	M	56y11m	Current	Parotid gland	SDC	
sd04	M	69y0m	No	Parotid gland	SDC	Squamous differentiation
sd05	M	57y9m	Current	Parotid gland	SDC	
sd06	M	71y4m	Ex	ubmandibular glar	SDC	
sd07	M	65y2m	?	Parotid gland	SDC	
sd08	M	46y9m	No	Parotid gland	SDC	
sd09	M	80y6m	?	ubmandibular glar	SDC	Hybrid with adenoid cystic carcinoma
sd10	M	49y5m	Current	Parotid gland	SDC	

Continued

Number	Tumor.size	T classification	N classification	Meta.LN/Total	Lymphatic_inv	Perivascular_inv	Perineural_inv	M at diagnosis
sd01	1.7x1.5 cm	T3	N0	0	N	N	N	M0
sd02	4.5x3 cm	T4a	N2b	63/67	Y	N	Y	M0
sd03	0.3x0.2 cm	T2	N0	0	N	N	N	M0
sd04	3.6x3.2x3 cm	T3	N2b	0	N	N	N	M0
sd05	2.8x2.7x2.3 cm	T2	N2b	13/20	Y	Y	N	M0
sd06	2x1.5 cm	T3	N0	0	N	N	Y	M0
sd07	1.6x1.2 cm	T1	N0	0	N	?	?	M0
sd08	1.5x1.3 cm	T4a	N0	0	N	N	Y	M0
sd09	2.2x1.9x1.0 cm	T4a	N0	7/20	Y	Y	Y	M0
sd10	2.1x1.7x0.6 cm	T2	N2b	22/26	Y	Y	Y	M0

Continued

Number	AR staining	Recurrence	Recurrence (mo)	Survival.status	Survival (mo)
sd01	Negative	N		NED	60
sd02	Positive	Y	20	DOD	40
sd03	Positive	N		NED	32
sd04	Positive	N		NED	28
sd05	Positive	Y	12	DOD	24
sd06	Positive	Y	11	AWD	27
sd07	Positive	N		NED	44
sd08	Positive	Y	36	AWD	73
sd09	Negative	Y	15	DOD	17
sd10	Positive	Y	12	AWD	15

Supplementary Table 3: Recurrently mutated genes

Gene	nnon	npat
TP53	12	12
RB1	7	7
SMAD4	12	7
APC	7	7
PIK3CA	6	5
HRAS	4	4
GNAQ	4	4
ALK	4	4
ERBB2	3	3
FLT3	3	2
ERBB3	2	2
ERBB4	2	2
MET	3	2

Non-silent recurrent mutations identified from whole-exome sequencing and targeted-sequencing were summarized as above. Shown in the table are their gene names, number of non-silent mutations (nnon) identified from the cohort and number of independent patients harboring such mutations (npat).

Supplementary Fig. 1: Classic example of salivary duct carcinoma. (A) Duct carcinoma with comedonecrosis (B) Invasive carcinoma area (C) Positive immune-reactivity with androgen receptor

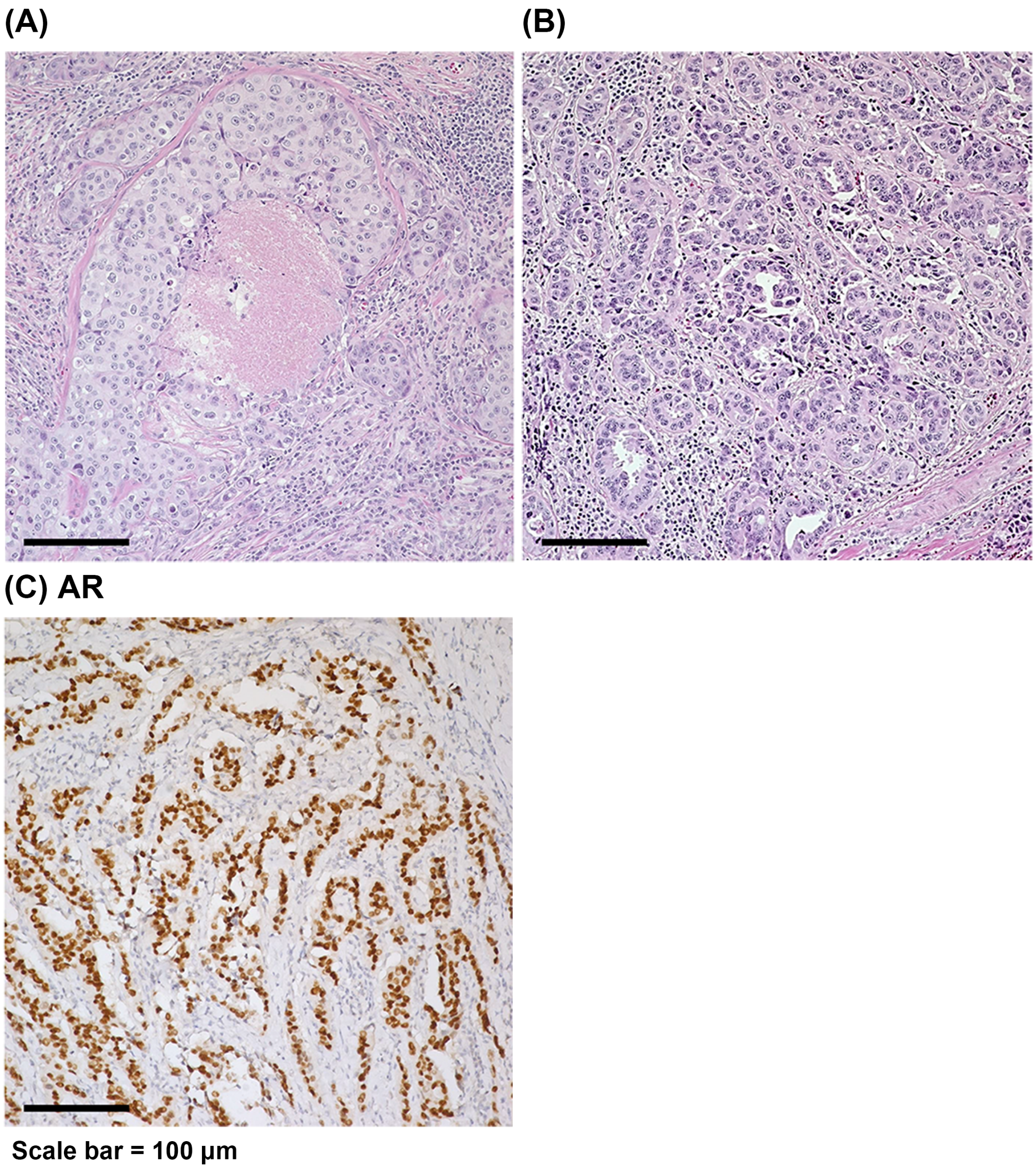

Supplementary Fig. 2: Clustering of SDC mutation signature

Unbiased hierarchical clustering of contributing weights of each mutational signature to the samples in the current study yielded three mutation signature clusters. The violet cluster in the middle commonly possessed signature 3, which was associated with BRCA-related mutational processes. The dark orange cluster was characterized by signature 16. APOBEC signatures (signature 2,13) were more broadly interspersed between the clusters. An outlying sample (sd01) consisted of another signature cluster. This sample displayed a low rate of somatic mutation with a distinct mutational process implicated.

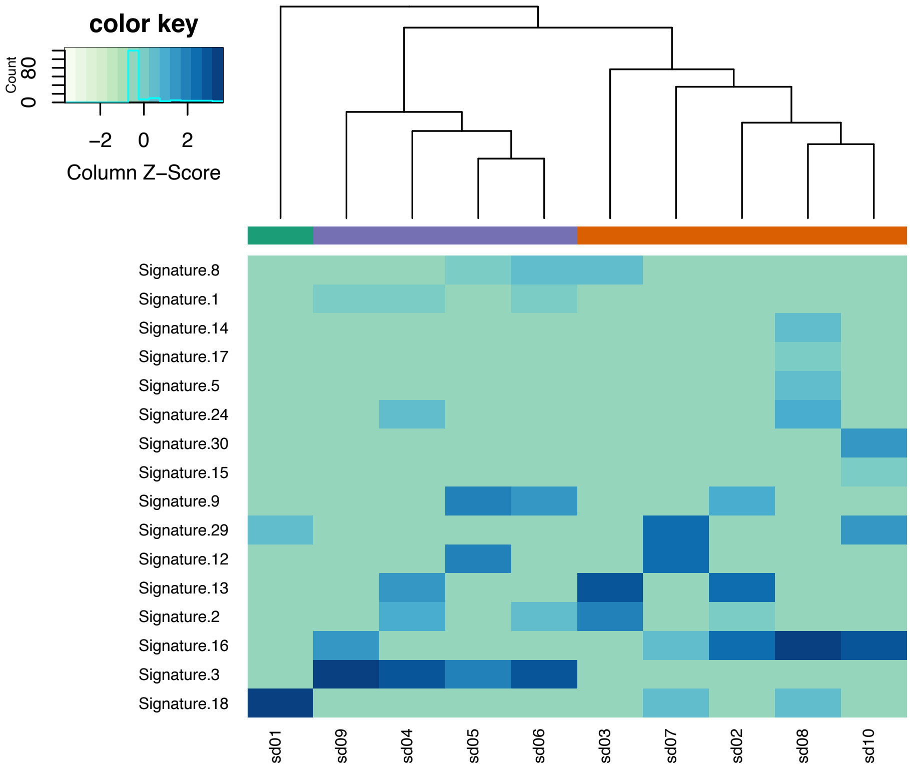

Left column represents signatures contributing to the current SDC cohort, bottom row represents patients' sample ID and color key represents sample-wise normalized representation of contributing mutational signatures.

Supplementary Fig. 3: Rainfall plot of inter-mutational distances between somatic single-base substitutions of sd04 sample. X-axis is the location on the genome segregated by chromosome, lower plot y-axis is inter-mutational distances in log10 scale, upper plot y-axis is the density of somatic single-base substitutions. Two genomic loci, including chr1 and chr11, demonstrated highly clustered somatic mutations.

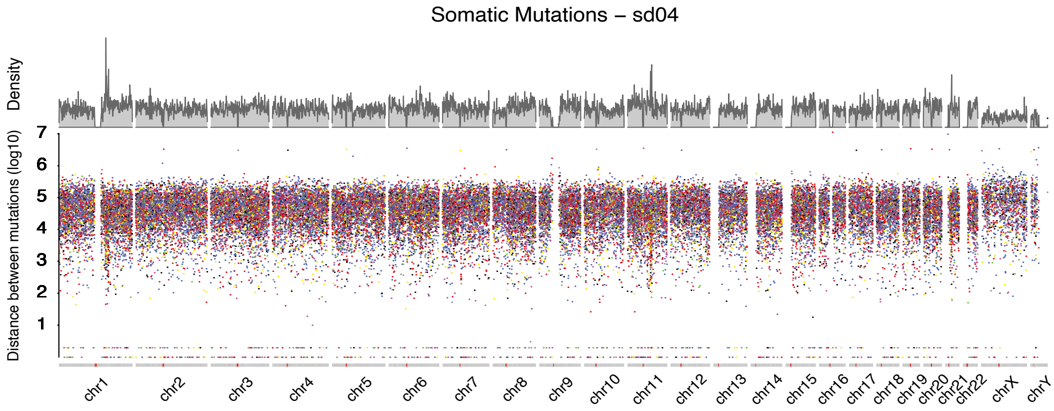

Supplementary Fig. 4: Identification of somatic copy number alterations in each SDC samples.

Whole genome sequencing data of each of SDC matched pairs were used to perform analysis of somatic copy number alterations. Depicted below is the copy number representation from FACETS algorithm. X-axis is the chromosomal number and y-axis is ploidy and absolute copy number estimation.

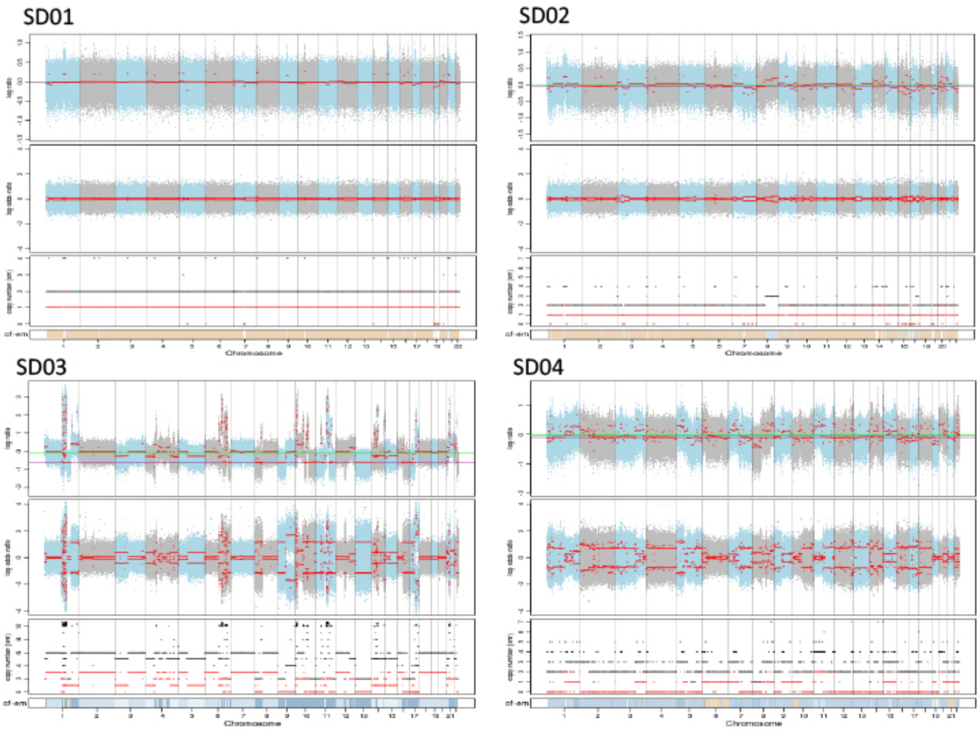

SD05

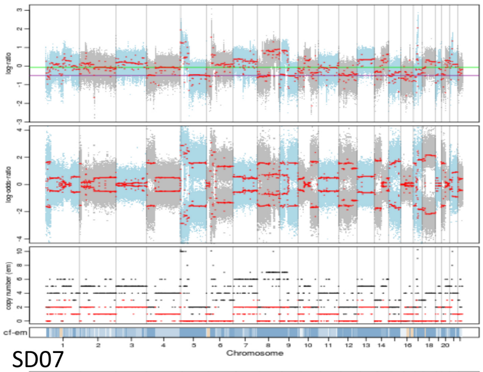

SD06

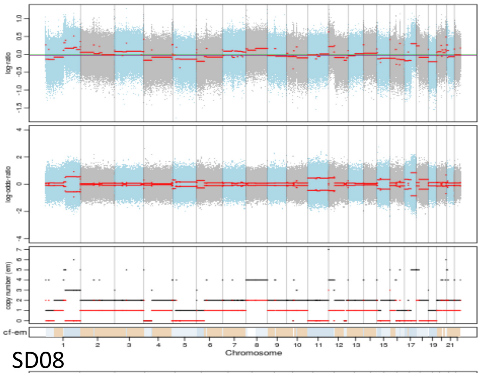

SD07

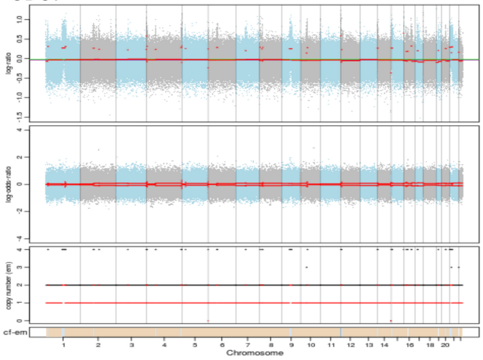

SD08

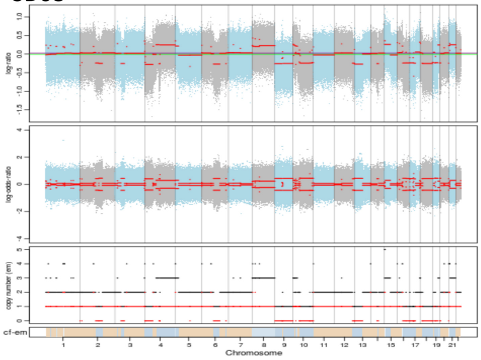

SD09

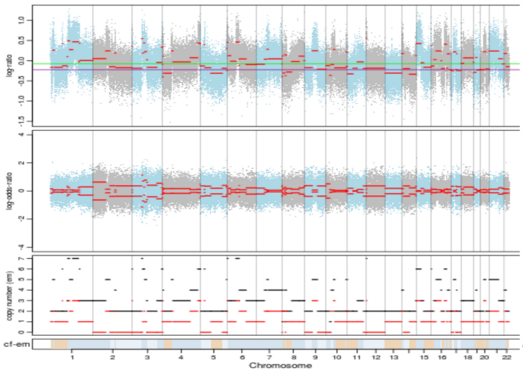

SD10

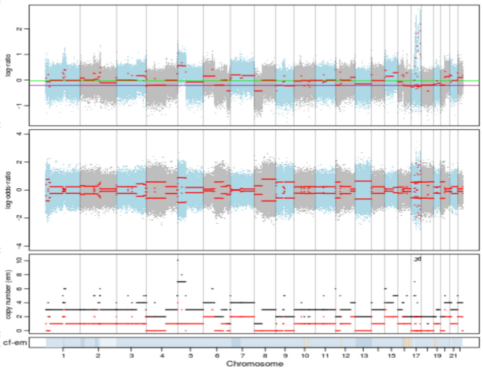

Supplementary Fig. 5: Statistical annotation of recurrently amplified/deleted genomic regions in SDC cohort

The segmentation output from FACETS/BIC-seq2 was used as an input file to GISTIC 2.0 analyses. GISTIC false discovery rate (FDR) q-values (x-axis) are plotted across genomic and chromosomal location.

Shown red are chromosomal region with focal amplifications, and depicted blue are those with focal deletions. Chromosomal Region are marked with peaks that exceed certain q-values.

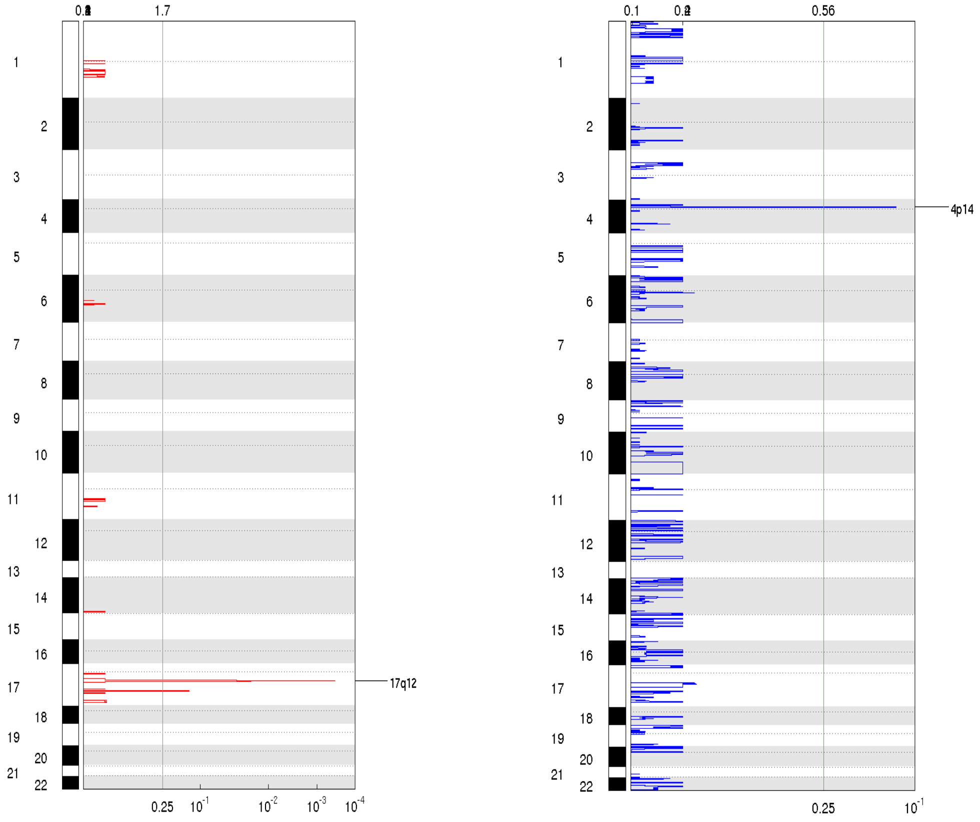

(a) amplification (b) deletion

Regions of focal copy number amplification detected by GISTIC 2.0 are annotated in the supplementary data file. Listed in the file are the names of the genes within the peak regions of copy number alterations.

Supplementary Fig. 6: Inactivation of *CDKN2A* gene by DNA rearrangement in a SDC sample.

DNA structural rearrangement study has identified a case (sd05) involving DNA rearrangement and inactivation of *CDKN2A* gene. Intronic region of *CDKN2A* between 1st and 2nd exone of the gene is structurally rearranged with intergenic region of *RP11-656G20.1-RNA5SP271*. Figure was generated with RCircos package.

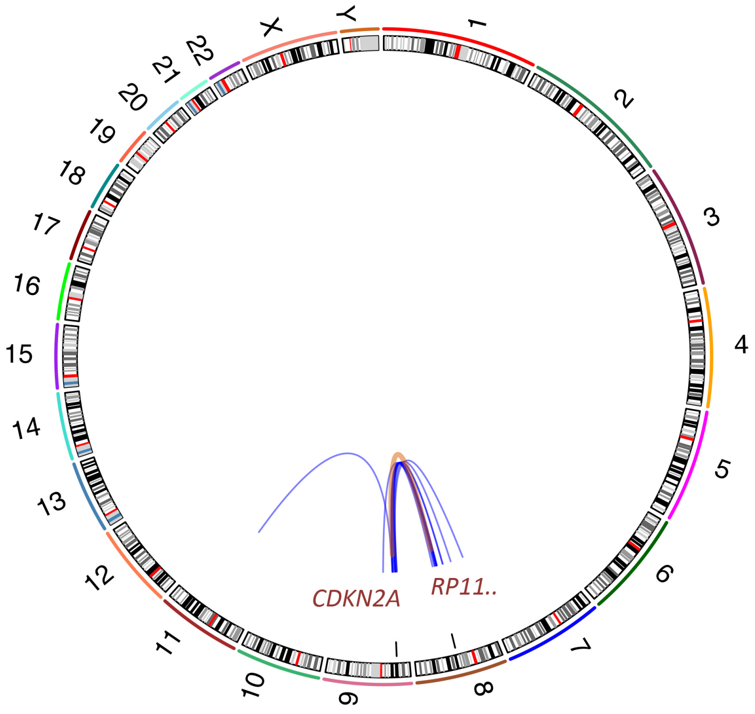

Supplementary Fig. 7: Overexpression of *MYB, NHSL1* gene in the sample containing *MYB-NHSL1* fusion

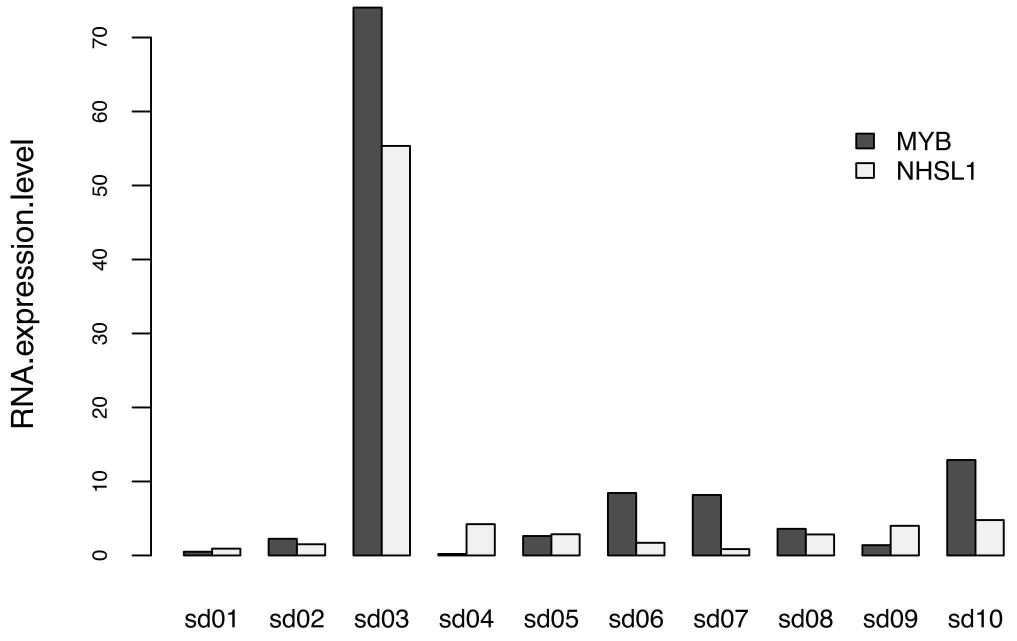

Expression level of *MYB* and *NHSL1* in the current SDC cohort demonstrated that a sample (sd03) containing *MYB-NHSL1* fusion gene displayed concomitant over-expression of both transcripts, compared to other non *MYB-NHSL1* samples. Y-axis is normalized RNA expression level from transcriptome sequencing data.

Supplementary Fig. 8: Pathological review of SDC sample containing *MYB-NHSL1* fusion gene

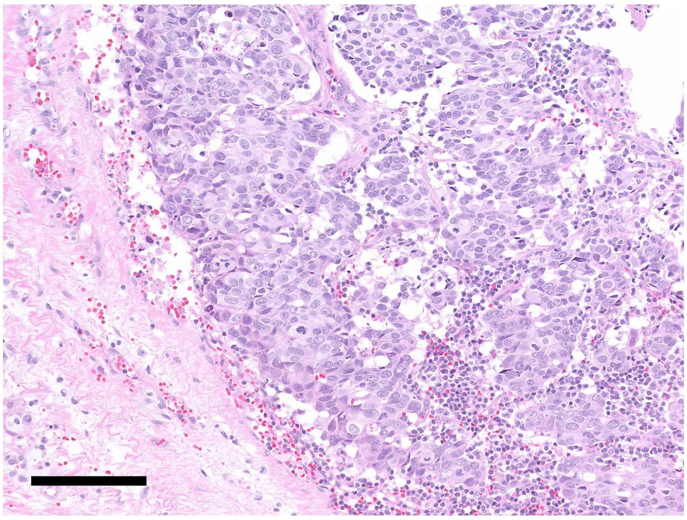

(A) Hematoxylin-eosin staining of the sample containing *MYB-NHSL1* fusion gene demonstrated the presence of ductal carcinoma in the tumor sample. Scale bars indicate 100 µm.

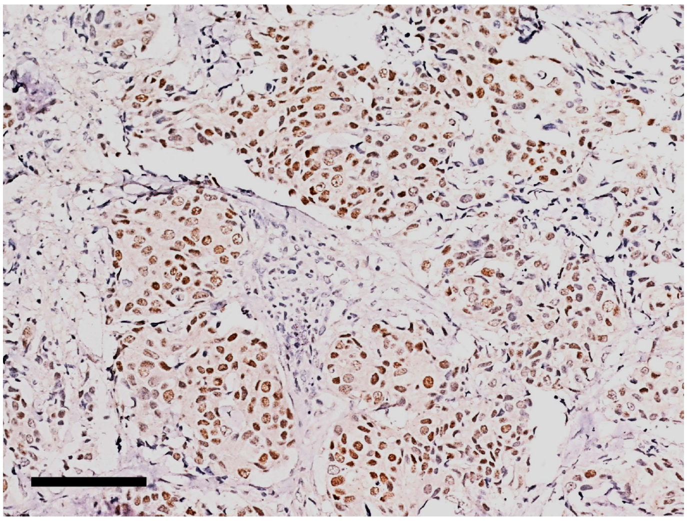

(B) The same sample containing the fusion gene showed positive immuno-staining of androgen-receptor in its carcinoma compartment. Scale bars indicate 100 µm.

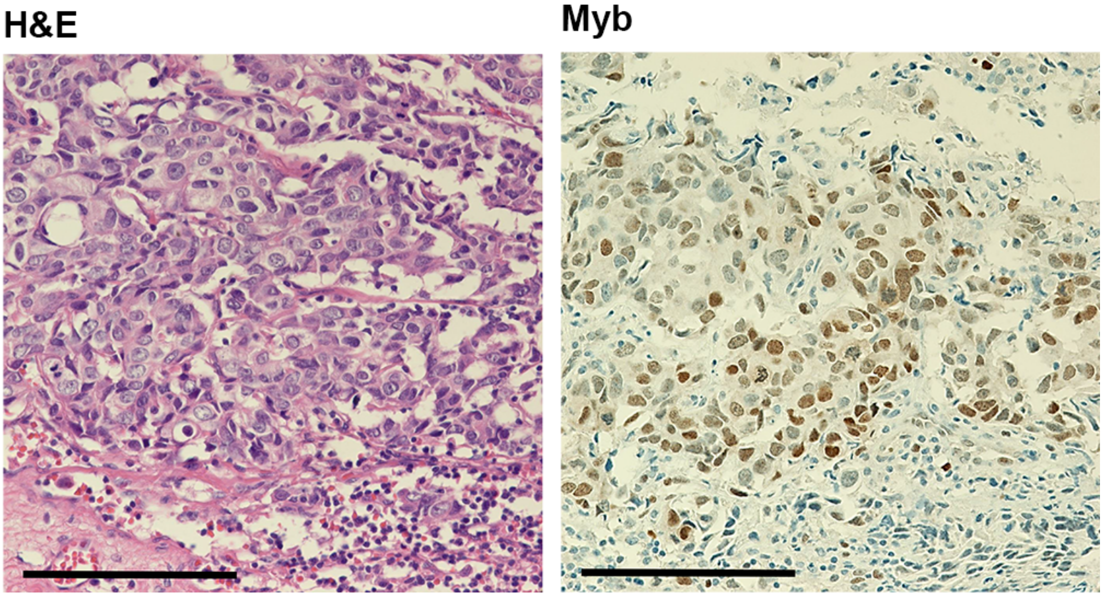

(C) The same sample containing the fusion gene showed positive immuno-staining of Myb protein in their nucleus.

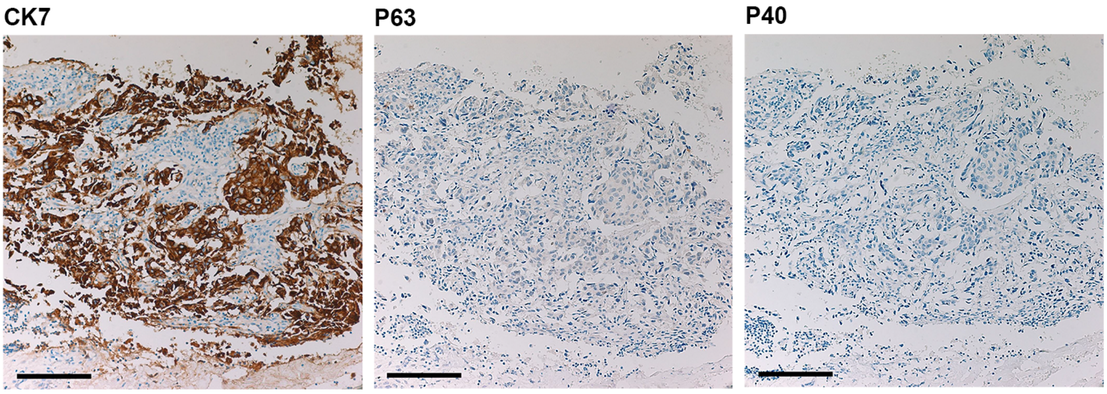

(D) Immunostainging for CK7, P63 and P40

Supplementary Fig. 9: Immune-profiling of SDC tumor samples

Tumor RNA sequencing data was used to de-convolute the profile of immune cells in SDC surgical tissue samples. Shown below is the relative composition of various immune cells in tumor compartment. Shown on the right legend is the colored definition of immune compartment that was used to de-convolute the immune composition in SDC samples.

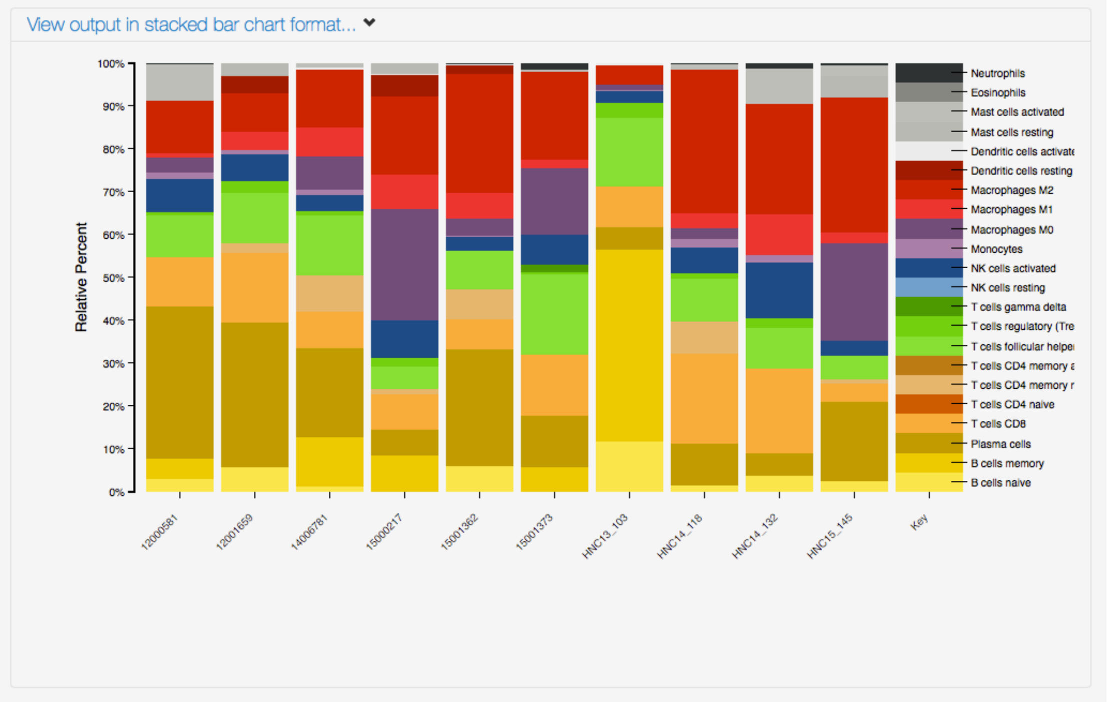

Supplementary Fig. 10: Tumor-map representation of SDC

RNA sequencing data was processed following the processing pipeline of TCGA data. In the updated pan-can tumour map, the RNA transcriptome of each sample was mapped according to their RNA expression profile and represented in 2-D principal component analysis plot. The most closely matched tumour clusters in the pan-can tumour map space was matched with each samples' RNA sequencing data. Shown below is the tumor map representation of 10 SDC transcriptome samples. Red balloon marks represent each of 10 SDC samples. 8 out of 10 SDC samples were closely clustered with breast cancer subtypes (represented in pink in the upper left corner of the figure below).

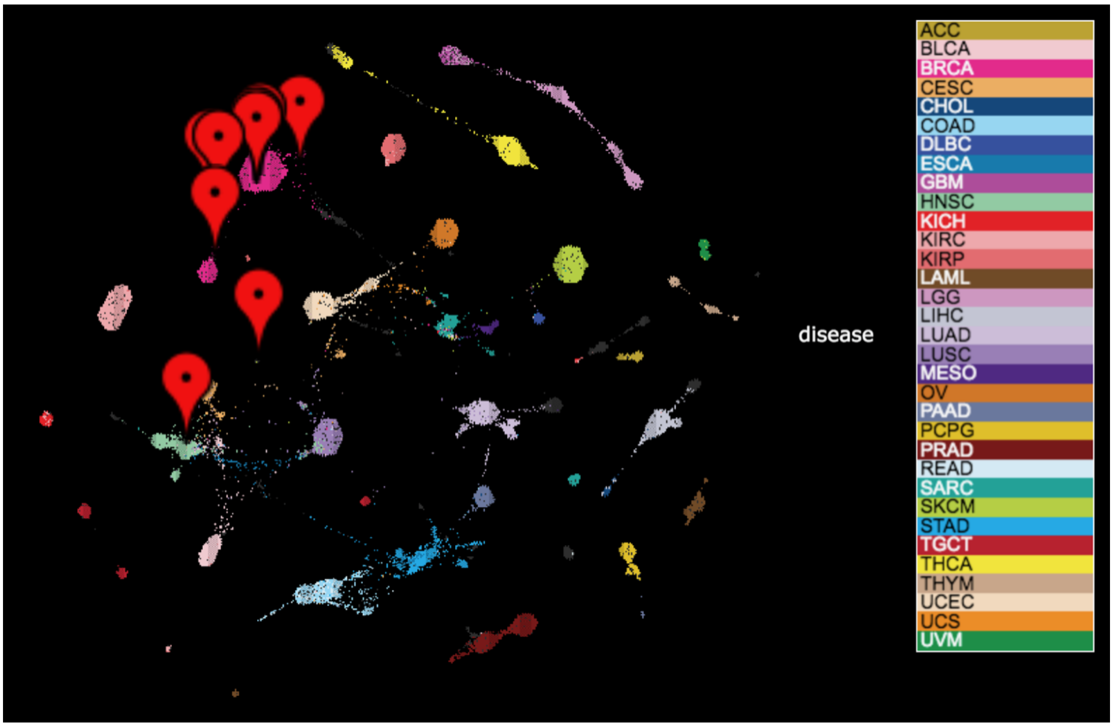

Two samples were not co-clustered with BRCA (one orphan assignment and the other in the head and neck squamous cell cancer, pale blue in the figure above). Histological examination of these two samples indicated slight squamous differentiation, reflecting their assignment out of breast cancer attribute.

Shown below is the enlargement of breast cancer tumor map clusters. Co-clustered with Her2 subtype of BRCA were 3 SDC samples, two out of which displayed ERBB2 focal somatic copy number alterations (3 red balloon marks in the yellowish tail). 4 other samples were co-clustered with LumA subtype (blue cluster), where 1 sample was co-segregated with Basal subtype of BRCA.

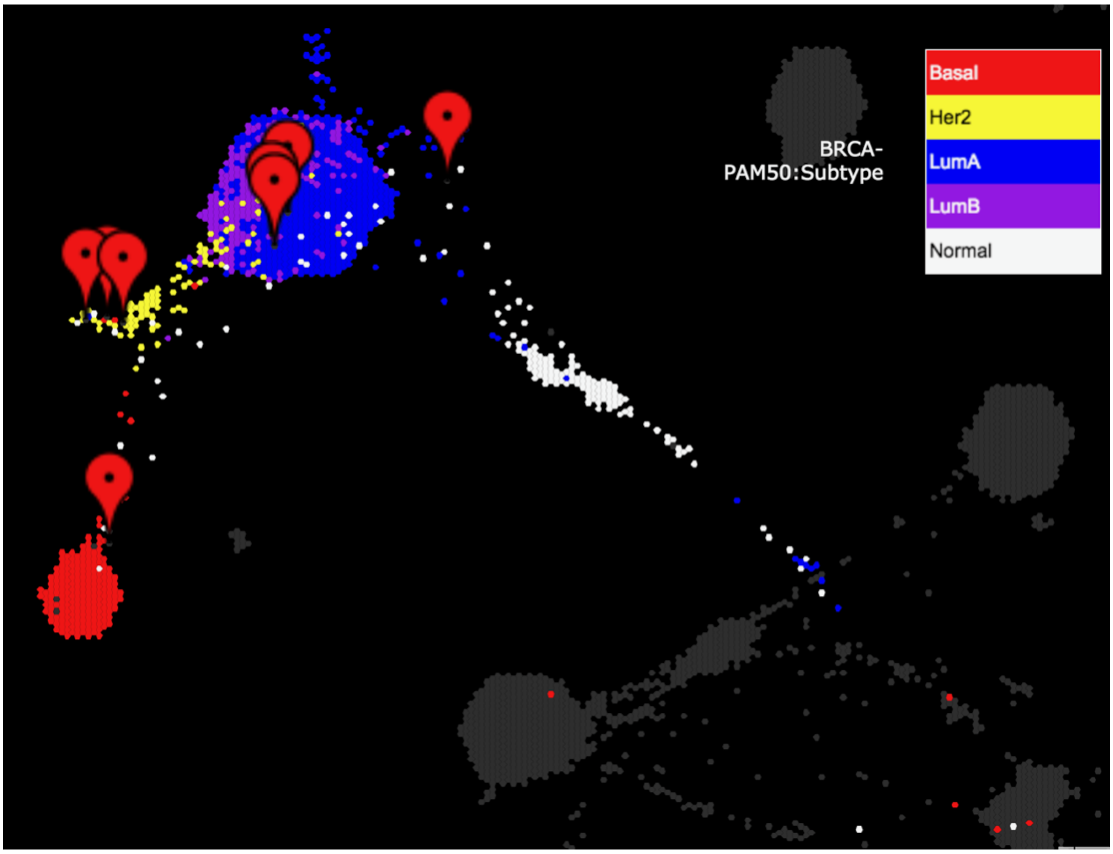

Supplementary Fig. 11: Clinical association with somatic mutations and patients' overall survival

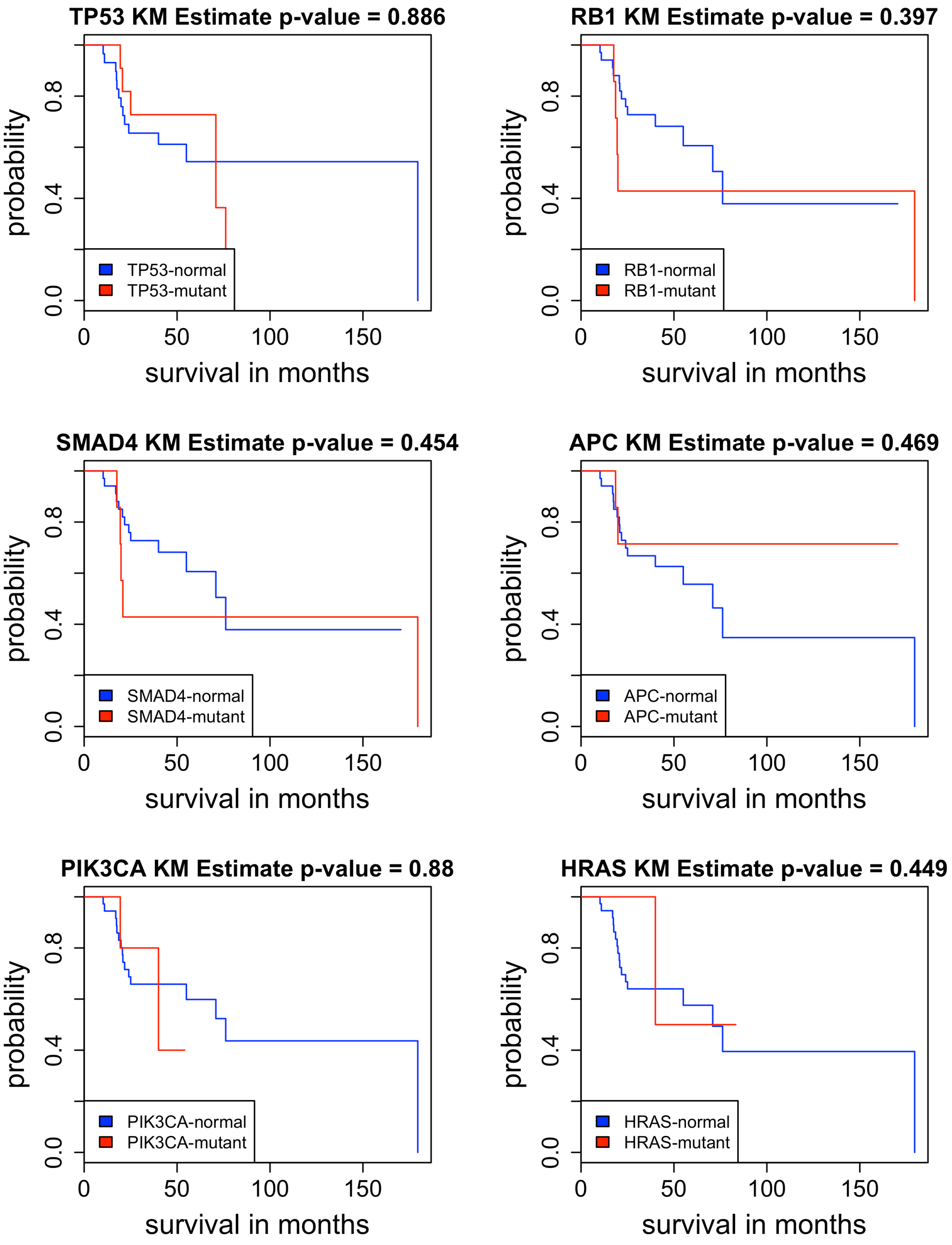

Blue lines indicate patients with wild-type of represented genes, and red lines indicate patients with mutations in the gene. In the title of each figure, the name of genes analyzed and their p-value obtained from survival analysis is indicated. The survival analysis was done with log-rank test.

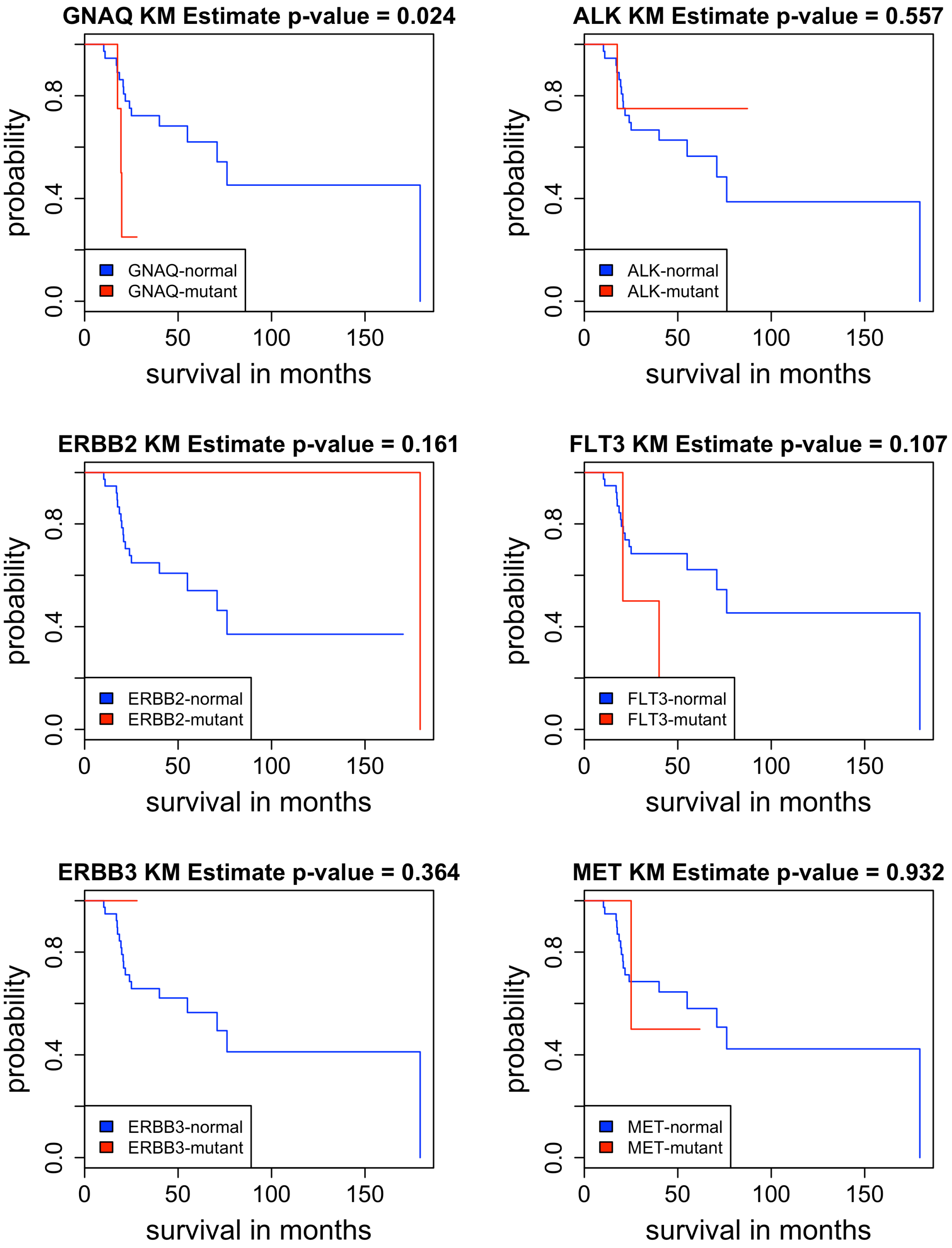
GNAQ KM Estimate p-value = 0.024
GNAQ-normal
GNAQ-mutant
ALK KM Estimate p-value = 0.557
ALK-normal
ALK-mutant
ERBB2 KM Estimate p-value = 0.161
ERBB2-normal
ERBB2-mutant
FLT3 KM Estimate p-value = 0.107
FLT3-normal
FLT3-mutant
ERBB3 KM Estimate p-value = 0.364
ERBB3-normal
ERBB3-mutant
MET KM Estimate p-value = 0.932
MET-normal
MET-mutant
probability
survival in months
0.0
0.4
0.8
0
50
100
150

Supplementary Fig. 12: Clinical association with somatic copy number alterations

Red lines indicate SDC patients with somatic copy number alterations in the corresponding genes, and blue lines indicate patients with diploid state of the corresponding genes. In the title of each figure, the name of genes analyzed and their corresponding p-values are shown. The p-values were obtained from log-rank test.

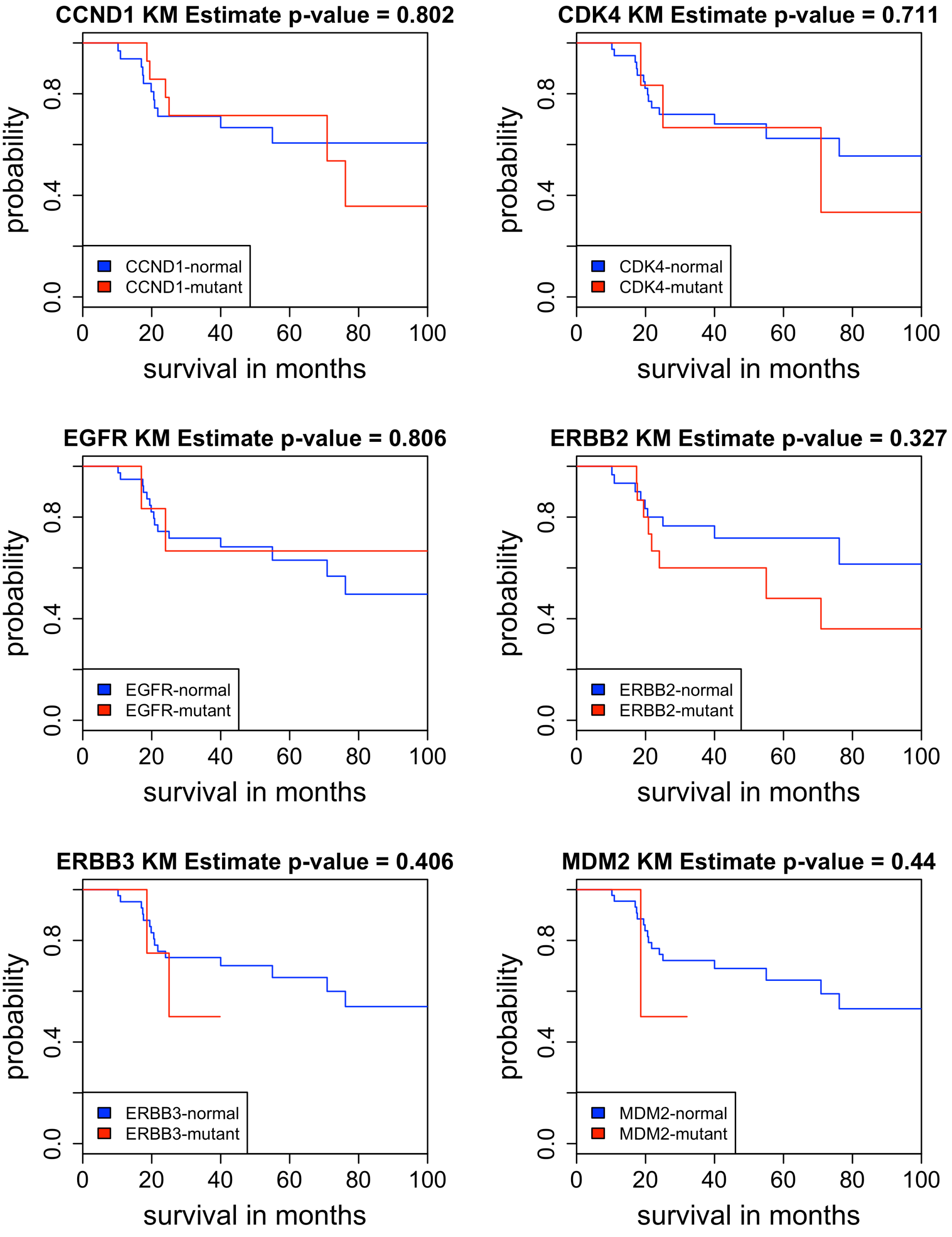

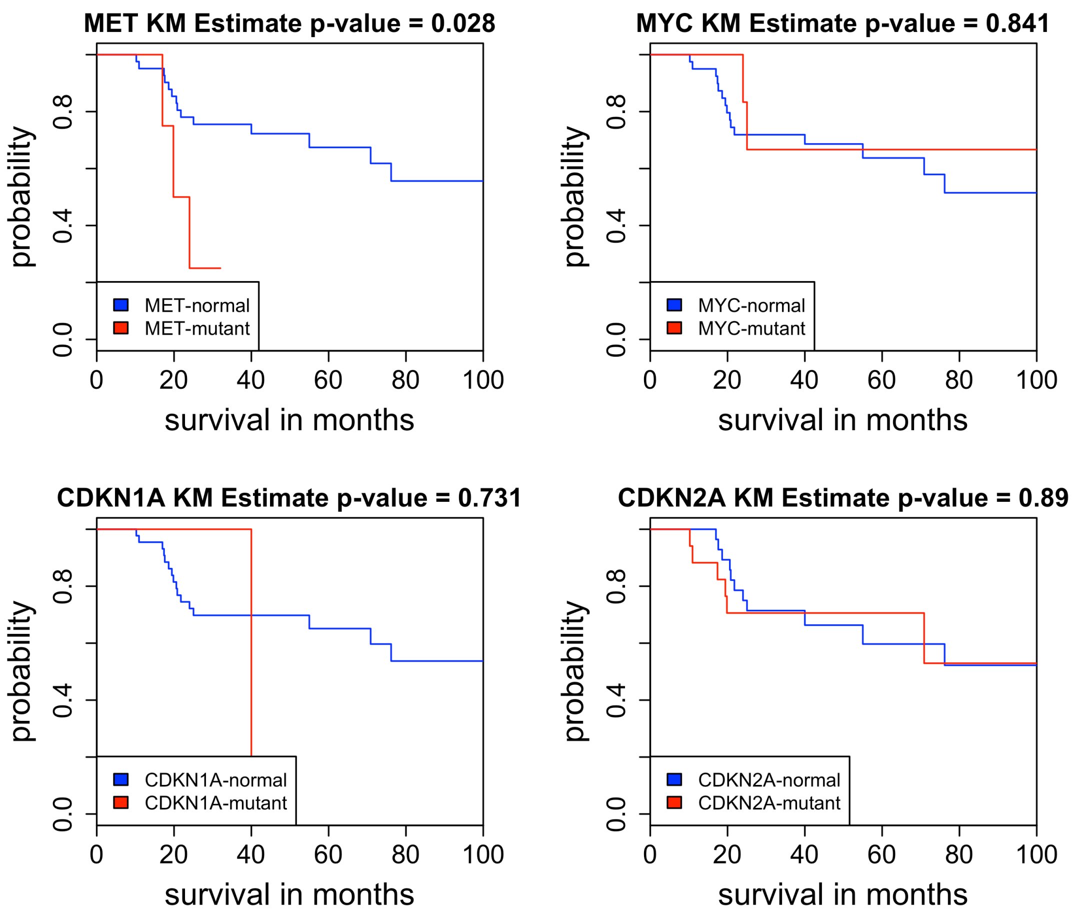

The statistical association of major genetic alterations including mutations and focal SCNAs with overall survival was examined by computing Harvard Ratio (HR) using the Cox proportional hazards regression model implemented into 'coxph in the R survival packages, 'survival'.

Kaplan-Meier curves (Supplementary Figs.11-12) were generated with 'survival' R package and p-valued were annotated by the log-rank test for right-censored survival data defined as the interval from the date of initial surgical resection to the date of last known contact or patient's death. Type 1 error rates were adjusted by calculating q-values in multiple hypothesis testing.

[END]
